# Functional versus functional and anatomical criteria-guided ranibizumab treatment in patients with neovascular age-related macular degeneration – results from the randomized, phase IIIb OCTAVE study

**DOI:** 10.1186/s12886-019-1251-6

**Published:** 2020-01-09

**Authors:** Giovanni Staurenghi, Justus G. Garweg, Bianca S. Gerendas, Wayne Macfadden, Boris Gekkiev, Philippe Margaron, Cornelia Dunger-Baldauf, Petr Kolar

**Affiliations:** 1 Department of Biomedical and Clinical Sciences “Luigi Sacco”, Sacco Hospital, University of Milan, G. B. Grassi, 74, 20157 Milan, Italy; 2 Berner Augenklinik am Lindenhofspital and University of Bern, Bern, Switzerland; 3 Vienna Reading Center, Department of Ophthalmology and Optometry, Medical University of Vienna, Waehringer Guertel 18–20, 1090 Vienna, Austria; 4 International Psychiatric Services, 1900 JFK Boulevard, #621, Philadelphia, PA 19103 USA; 5 Novartis Pharma AG, Postfach, 4002 Basel, Switzerland; 6 Slowak Medical University and University Hospital Bratislava, Antolska 11, 85107 Bratislava, Slovakia

**Keywords:** Best-corrected visual acuity, Neovascular age-related macular degeneration, Optical coherence tomography, Ranibizumab, Retreatment criteria

## Abstract

**Background:**

To evaluate the efficacy and safety of two individualized ranibizumab retreatment schemes in neovascular age-related macular degeneration.

**Methods:**

Patients (*N* = 671) were randomized (1:1) to receive three initial monthly ranibizumab 0.5 mg injections, then retreatment guided by either best-corrected visual acuity (BCVA) loss (Group I) or BCVA loss and/or signs of disease activity on optical coherence tomography (OCT; Group II). The study was terminated prematurely and the decision to discontinue the study was made by the sponsor. Efficacy analyses were performed on patients who completed 12 months of the originally planned 24-month study. Safety analyses are presented for all safety analyzable patients.

**Results:**

Of 671 randomized patients, 305 completed 12 months of the study. For the 12-month completers, baseline mean (standard deviation) BCVA and reading-center evaluated central subfield thickness (CSFT) were comparable [Group I: 60.9 (13.10) letters and 517.7(201.79) μm; Group II: 60.2 (12.21) letters and 515.3 (198.37) μm]. The change from baseline at Month 12 in BCVA was 6.7 (13.48) letters in Group I and 8.3 (13.53) letters in Group II and the change in CSFT was − 161.3 (163.48) μm and − 175.3 (170.45) μm, respectively. The mean number of ranibizumab injections was 8.2 in Group I and 8.4 in Group II.

**Conclusion:**

Ranibizumab treatment resulted in visual and anatomic gains at 12 months for both retreatment strategies, with a trend in favor of OCT-guided vs BCVA loss guided retreatment. No new safety signals were seen.

**Trial registration:**

www.ClinicalTrials.gov (NCT01780935). Registered 31 January 2013.

## Background

Anti-vascular endothelial growth factor (anti-VEGF) agents are the standard of care for the treatment of neovascular age-related macular degeneration (nAMD) [1, 2]. Ranibizumab, an anti-VEGF antibody fragment specifically designed for ophthalmic use [3], is approved for the treatment of nAMD in the United States [4], Europe [5] and many other countries worldwide.

With the increasing use of optical coherence tomography (OCT), evidence emerged that anatomical changes may precede VA loss and therefore, could be used as an early indicator for retreatment decisions [6–10]. In the CATT and HARBOR studies, predominantly OCT-based retreatment decisions resulted in patient outcomes comparable to those seen with monthly dosing [6, 7, 9]. The PrONTO study demonstrated the usefulness of OCT for guiding retreatment with intravitreal ranibizumab in nAMD [8, 10]. A *post-hoc* analysis of the EXCITE study demonstrated that patients with intraretinal cystoid fluid may require more injections than others to maintain vision [11].

The OCT And Vision Evaluation (OCTAVE) study (NCT01780935) [12] was designed to assess the efficacy and safety of two ranibizumab 0.5 mg treatment regimens, with retreatment decisions guided by functional (VA) versus functional and/or anatomical (VA and/or OCT-guided) criteria, in patients with nAMD. Prior to this study, no prospective study had compared patient outcomes using a retreatment strategy based on VA loss alone with one that permitted retreatment if anatomical signs of disease activity were also observed. However, after the OCTAVE study was initiated, the use of OCT in retreatment decisions was widely accepted by ophthalmologists and health authorities [13, 14]. After a careful and thorough review of the study objectives, the decision to terminate OCTAVE early was taken. This was considered to be in the best interest of patients in the study for whom the retreatment strategy was based on VA loss alone. Nevertheless, the data collected provide valuable additional information on ranibizumab retreatment criteria in patients with nAMD. Here, we describe results from an efficacy and imaging analysis performed on patients who completed 12 months of the OCTAVE study. Safety analyses are presented for all safety-analyzable patients.

## Methods

### Study design

**Fig. 1:**
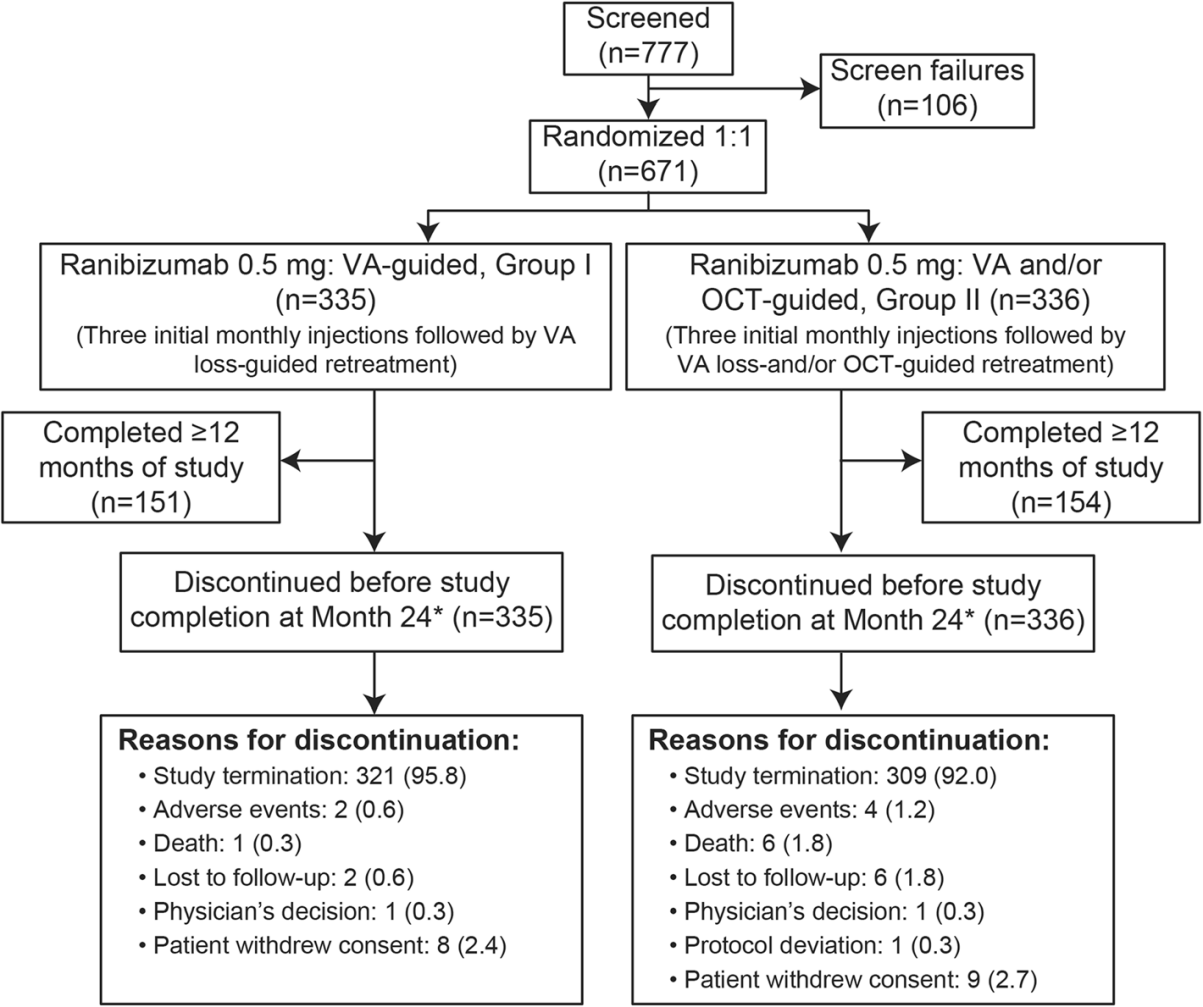
Patient disposition. The reasons for screening failure were: patients did not meet the diagnostic or severity criteria. (44 patients [41.5%]), unacceptable test procedure results (20 patients [18.9%]), other (20 patients [18.9%]), and patients’ withdrawal of consent (19 patients [17.9%]). *The study was prematurely terminated due to the emergence of OCT as an important technique for diagnosis and making treatment/retreatment decisions in nAMD; none of the patients completed 24 months. nAMD, neovascular age-related macular degeneration; OCT, optical coherence tomography; VA, visual acuity

OCTAVE was an open-label, phase IIIb, randomized, double-masked (with regard to the retreatment criteria) study that was originally planned to have a 24-month duration (Fig. 1), but was terminated earlier than planned (October 2014). The decision to discontinue the study was made by the sponsor after OCT-guided monitoring of disease activity has been included in the posology for ranibizumab and due to the updated label approved for disease activity (including VA and anatomical parameters). The OCTAVE study was initiated in June 2013 and completed in July 2015. The study was conducted at 92 sites in 24 countries.

OCTAVE was conducted according to the principles of the Declaration of Helsinki and the study protocol was reviewed by the Independent Ethics Committee or Institutional Review Board for each study center (see Additional file 1: Table S1, which lists all the IECs/IRBs). Patients provided written informed consent. The manuscript reporting adheres to the CONSORT guidelines for the reporting of randomized trials.

### Randomization and treatment

Eligible patients were randomized (1:1) via Interactive Response Technology to either of the two treatment groups, using a validated system that automated the random assignment of patient numbers to randomization numbers: Group I (VA-guided): three initial monthly ranibizumab 0.5 mg injections with retreatment thereafter at the investigator’s discretion based on best-corrected visual acuity (BCVA) loss due to nAMD; Group II (VA and/or OCT-guided): three initial monthly ranibizumab 0.5 mg injections with retreatment thereafter at the investigator’s discretion based on BCVA loss due to nAMD and/or signs of nAMD disease activity on OCT. Both groups had the same monthly assessments, including OCT. The VA assessor was masked to the retreatment strategy assignment and performed only the BCVA assessment. The evaluating and/or treating investigator (who could be the same person) was not masked to the retreatment strategy and performed all other study related activities. The decision to retreat was solely at the investigator’s discretion as per the retreatment criteria. During the study, no rescue medication was permitted for the treatment of nAMD.

### Patients

Patients included were ≥ 50 years of age with visual impairment due to nAMD; with active, newly diagnosed, angiographically documented choroidal neovascularization (CNV) secondary to AMD in a previously untreated eye; and with CNV or its sequelae (i.e., pigment epithelium detachment, subretinal or subretinal pigment epithelial hemorrhage, blocked fluorescence, macular edema, or subretinal, subretinal pigment epithelial or intraretinal fluid [IRF]) involving the center of the fovea, and a BCVA score at both screening and baseline between 78 and 23 letters inclusive (approximate Snellen equivalent of 20/32 and 20/320). If both eyes were eligible for the study, a single study eye was selected at screening by the investigator based on clinical judgement and the decision was confirmed at the baseline visit.

Key exclusion criteria applied at screening or baseline were active periocular or ocular infection or inflammation; uncontrolled glaucoma (intraocular pressure [IOP] ≥ 30 mmHg on medication or according to the investigator’s judgment); neovascularization of the iris or neovascular glaucoma; cataract (if causing significant visual impairment), aphakia, vitreous hemorrhage, rhegmatogenous retinal detachment, proliferative retinopathy or CNV of any other cause than nAMD; irreversible structural damage within 0.5 disc diameter of the center of the macula (e.g., vitreomacular traction, epiretinal membrane, scar, laser burn, macular hole) that in the investigator’s opinion could substantially impact visual function improvement with treatment; atrophy or fibrosis involving the center of the fovea; and total area of fibrosis comprising more than 50% of the lesion area (see Additional file 2 for detailed exclusion criteria).

### Assessments

The following efficacy variables were assessed: mean change from baseline over 12 months in BCVA and central subfield thickness (CSFT); mean change from baseline at Month 12 in BCVA and CSFT categorized by presence at baseline of extrafoveal CNV lesions (defined by the Vienna Reading Center [VRC] as lesions where the CNV border was more than 200 μm away from the foveal centerpoint), subretinal fluid (SRF) or cysts, and by thickness of cysts in the central millimeter subfield; proportion of patients with a BCVA improvement of ≥5, ≥10, and ≥ 15 letters from baseline at Month 12; and proportion of patients with BCVA ≥73 letters (20/40 Snellen equivalent) at Month 12.

BCVA was assessed in the study eye at all visits in a sitting position using Early Treatment Diabetic Retinopathy Study (ETDRS)-like VA testing charts at an initial testing distance of 4 m or at 1 m (if it was not possible to assess at 4 m). OCT images were taken using different spectral-domain OCT devices certified for multicenter study use by the VRC. Each patient was assessed using the same machine throughout the study. The investigator used the information collected to assess the status of disease activity. The images taken monthly from screening to end-of-study visit were sent to the VRC for further evaluation. The captured images contained quantitative parameters (CSFT and the presence or absence of qualitative parameters (intraretinal cysts (further referred to as cysts; defined as round, minimally reflective spaces within the neurosensory retina [15]) and SRF (identified as a non-reflective space between the posterior boundary of the neurosensory retina and the retinal pigment epithelium/choriocapillaris reflection [15]). The CSFT represented the average retinal thickness between the Bruch’s membrane and the inner limiting membrane of the circular area within 1 mm diameter around the foveal center. Fluorescein angiography was performed in conjunction with color fundus photography. The investigator could use these images at his/her discretion to confirm their decision to retreat. The investigator also sent these images to the VRC. Efficacy data are summarized descriptively, except for the explorative BCVA analysis investigating the impact of the presence of baseline cysts. A linear regression model for change in BCVA from baseline to Month 12 was fitted, including treatment, presence of baseline cysts as factors, baseline BCVA as covariate and presence of baseline cysts/treatment as interaction term.

Treatment exposure was evaluated in both groups over the entire study period. Safety assessments included the type, frequency, relationship, and severity of ocular and non-ocular adverse events (AEs). Safety assessments were performed in the safety set which consisted of all patients who received at least one application of study treatment and had at least one post-baseline safety assessment. Safety data for all safety-analyzable patients are summarized descriptively.

## Results

As noted earlier, the study was discontinued early prior to any patient completing the original 2 year study period. Enrollment of the originally intended, entire study population (*N* = 671) was complete; however, at the time of termination, patients had participated for variable periods of time within the study. Before termination, 14 (4%) patients in Group I and 27(8%) patients in Group II had discontinued mostly due to withdrawal of consent (8[2.4%] in Group I and 9[2.7%] in Group II). Of the 671 enrolled patients (Group I [*n* = 335], Group II [*n* = 336]), 305 completed at least 12 months and had a VA measurement at M12 (Group I [*n* = 151] and Group II [*n* = 154], Fig. 1**).** All enrolled patients were included in the safety set, except for one patient in Group I for whom neither study drug was administered nor safety assessments performed.

Here, we present baseline and safety data for the entire population, but analyses of efficacy data only for patients who completed at least 1 year within the study, so as to compare groups with a comparable time period of exposure to treatment.

The mean age of enrolled patients was 74.6 years, and most were female (60.7%) and Caucasian (96.1%). Mean (standard deviation [SD]) IOP at baseline was 15.1 (2.8) mmHg. Baseline demographic and ocular characteristics were comparable between the treatment groups in the randomized set and between treatment groups in patients who completed 1 year of treatment (Table 1). The most common lesion type was occult with no classic component and CNV location was extrafoveal in approximately one-third of patients. Baseline imaging data (OCT, FA) were also comparable between the randomized set and those who completed 12 months of treatment (Table 1).

**Table 1:** Baseline demographics and disease characteristics

Characteristics	Ranibizumab 0.5 mg VA only (Group I)		Ranibizumab 0.5 mg VA and/or OCT (Group II)	
	Randomized set *n* = 335	12-month completers *n* = 151	Randomized set *n* = 336	12-month completers *n* = 154
Mean (SD) age, years	73.9 (7.9)	74.1 (8.10)	75.3 (7.9)	74.9 (7.9)
Gender, Female, n (%)	213 (63.6)	91 (60.3)	194 (57.7)	93 (60.4)
Race, Caucasian, n (%)	320 (95.5)	150 (99.3)	325 (96.7)	154 (100)
Mean (SD) BCVA, ETDRS letters	61.9 (12.84)	60.9 (13.10)	59.8 (12.64)	60.2 (12.21)
Mean (SD) CSFT, μm	500.9 (203.58)	517.7 (201.8)	512.7 (193.80)	515.3 (198.37)
Mean (SD) time since first diagnosis of nAMD, weeks	6.6 (41.74)	8.3 (57.40)	4.8 (15.65)	5.5 (20.87)
Mean (SD) central subfield volume on OCT, mm^3^	0.4 (0.16)	0.4 (0.16)	0.4 (0.15)	0.4 (0.16)
Presence of subretinal fluid on OCT, Yes, n (%)	257 (76.7)	126 (83.4)	270 (80.4)	132 (85.7)
Presence of fluid beneath the retinal pigment epithelium on OCT, Yes, n (%)	122 (36.4)	59 (39.1)	132 (39.3)	59 (38.3)
Presence of cysts on OCT, Yes, n (%)	150 (44.8)	77 (51.0)	174 (51.8)	78 (50.6)
Presence of extrafoveal CNV lesions on FA, Yes, n (%)	123 (36.7)	56 (37.1)	108 (32.1)	53 (34.4)
Lesion type on FA, n (%)				
- 100% classic	73 (21.8)	40 (26.5)	72 (21.4)	35 (22.7)
- Predominantly classic	20 (6.0)	7 (4.6)	27 (8.0)	14 (9.1)
- Minimally classic	36 (10.7)	18 (11.9)	33 (9.8)	16 (10.4)
- Occult with no classic component	123 (36.7)	61 (40.4)	114 (33.9)	55 (35.7)

Randomized set comprises all randomized patients

*BCVA* best-corrected visual acuity, *CSFT* central subfield thickness, *ETDRS* Early Treatment Diabetic Retinopathy Study, *FA* fluorescein angiography, *n* number of patients in treatment group with Month 12 VA (for 12-month completers), *nAMD* neovascular age-related macular degeneration, *OCT* optical coherence tomography, *SD* standard deviation, *VA* visual acuity

### Efficacy outcomes

**Fig. 2:**
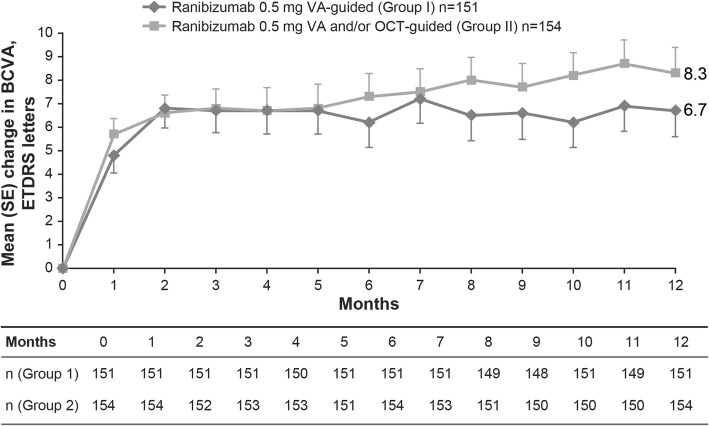
Mean change in BCVA from baseline up to Month 12. For Group I, error bars are shown in the negative direction and for Group II, error bars are shown in the positive direction. BCVA, best-corrected visual acuity; ETDRS, Early Treatment Diabetic Retinopathy Study; n, number of patients with Month 12 BCVA data; OCT, optical coherence tomography; SD, standard deviation; VA, visual acuity

**Fig. 3:**
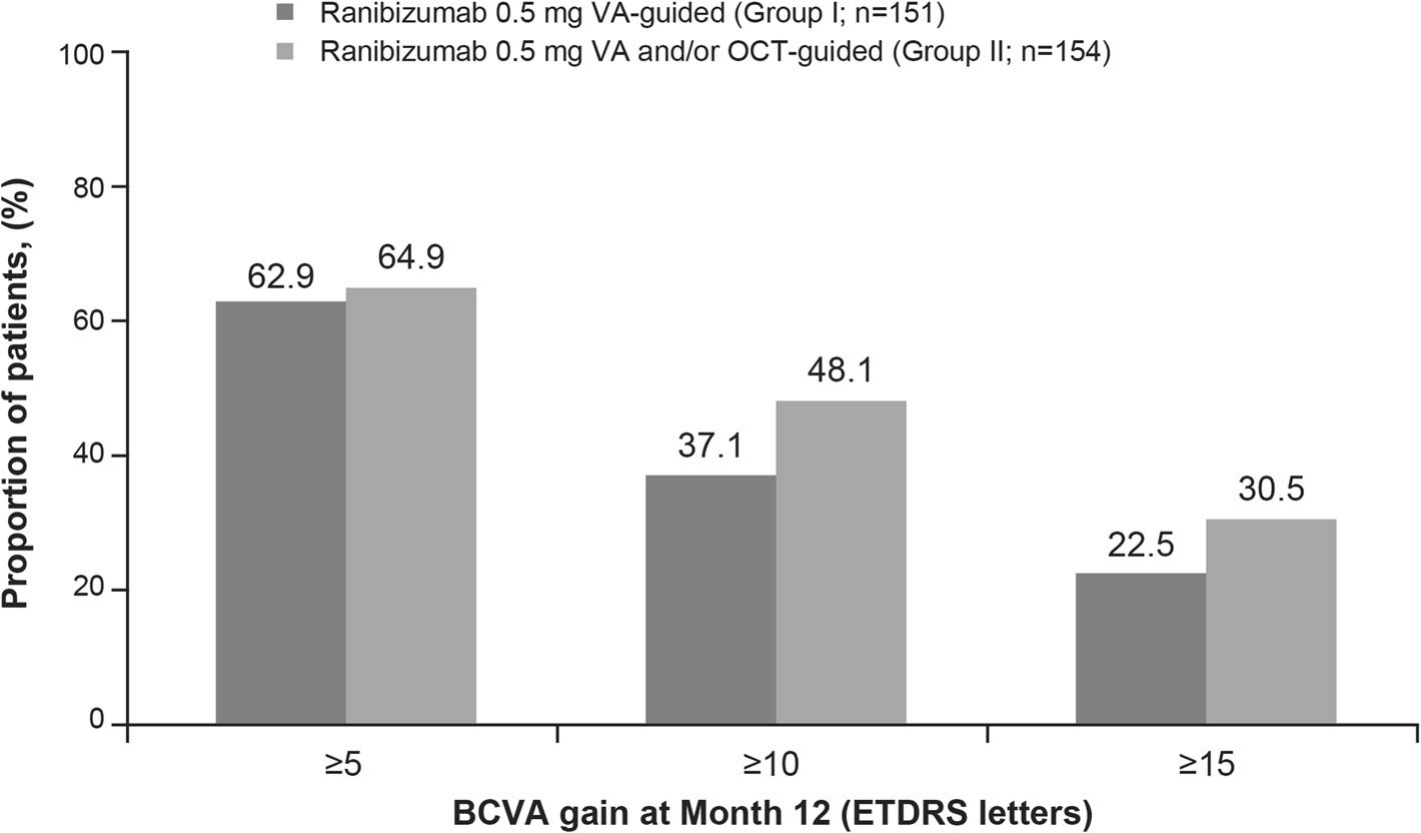
Proportion of 5, 10 and 15 letter gainers at Month 12. BCVA, best-corrected visual acuity; ETDRS, Early Treatment Diabetic Retinopathy Study; n, number of patients with Month 12 BCVA data; OCT, optical coherence tomography; VA, visual acuity

**Fig. 4:**
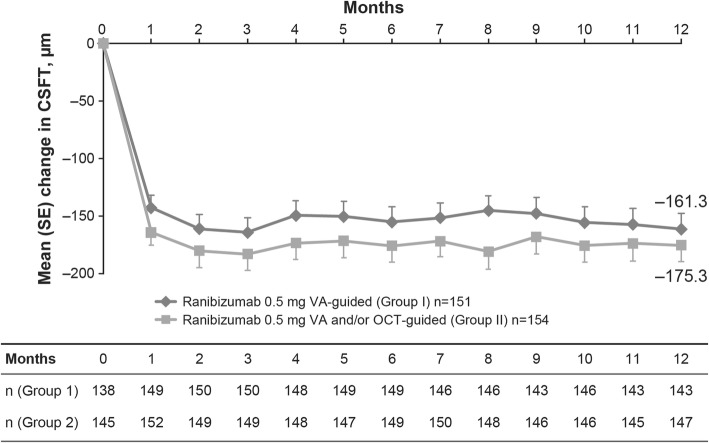
Mean change in CSFT from baseline up to Month 12. For post-baseline visits, baseline is defined as the last available non-missing value collected just prior to the start of treatment. For Group I, error bars are shown in the positive direction and for Group II, error bars are shown in the negative direction. CSFT, central subfield thickness; n, number of patients with Month 12 BCVA data; OCT, optical coherence tomography; VA, visual acuity

Mean (SD) BCVA increased from baseline to Month 12 (Fig. 2). At Month 12, mean (SD) gain was numerically lower in Group I compared with Group II (6.7 [13.48] vs 8.3 [13.53] letters; Fig. 2). BCVA gain of ≥10 or ≥ 15 letters at Month 12 was observed in both groups, but there was a trend towards more patients in Group II versus Group I achieving these gains (Fig. 3). Similarly, a numerically higher proportion of patients in Group II achieved BCVA ≥73 letters at Month 12 (54% vs 49%) than Group I. The mean (SD) CSFT decreased from baseline over time (Fig. 4) and at Month 12 was − 161.3 (163.48) μm in Group I and − 175.3 (170.45) μm in Group II.

### Effect of extrafoveal CNV lesions, SRF, cysts, and thickness of cysts at baseline on BCVA and CSFT outcomes

In both groups, there were differences in baseline BCVA and CSFT between patients with extrafoveal CNV lesions versus those with foveal (centerpoint is occupied by CNV) and juxtafoveal (border of CNV is 1–200 μm away from centerpoint) CNV lesions. There was a trend towards higher mean (SD) BCVA gains in patients with foveal and juxtafoveal lesions than those with extrafoveal CNV lesions in both groups; the gains were numerically higher in Group II than in Group I (see Additional file 3: Figure S1,). Mean (SD) decrease in CSFT from baseline at Month 12 was observed irrespective of the presence or absence of extrafoveal CNV lesions at baseline; the reduction was numerically higher in Group II in those with extrafoveal CNV lesions (see Additional file 3: Figure S1). In both groups, the mean change in BCVA or CSFT from baseline at Month 12 did not differ based on the presence or absence of SRF at baseline; the change was numerically higher in Group II irrespective of SRF (see Additional file 4: Figure S2).

Patients with cysts had a lower BCVA at baseline that stayed low over time compared with BCVA in patients without cysts at baseline, exploratory *p* < 0.05 for the difference in BCVA gain between presence/absence of cysts in both groups (linear regression, see**,** Additional file 5: Table S2**)**. The presence of cysts at baseline was associated with slightly lower BCVA gain from baseline at Month 12 in Group I versus Group II, whereas in those without baseline cysts, the BCVA gains from baseline at Month 12 were similar between both groups (see, Additional file 4: Figure S2). The difference in mean BCVA change from baseline to Month 12 by presence or absence of cysts at baseline was higher in Group I compared with Group II (7.64 vs 4.80 letters), as estimated by linear regression. The mean CSFT decrease from baseline to Month 12 was higher in both groups in patients with cysts at baseline than those without. The absolute reduction in CSFT was comparable between groups in those with baseline cysts and was numerically higher in Group II in those without baseline cysts (see, Additional file 4: Figure S2). The BCVA gain from baseline at Month 12 and reduction in mean CSFT from baseline at Month 12 were higher in both groups in those with cysts larger than 400 μm at baseline (see, Additional file 4: Figure S2).

### Treatment exposure

**Fig. 5:**
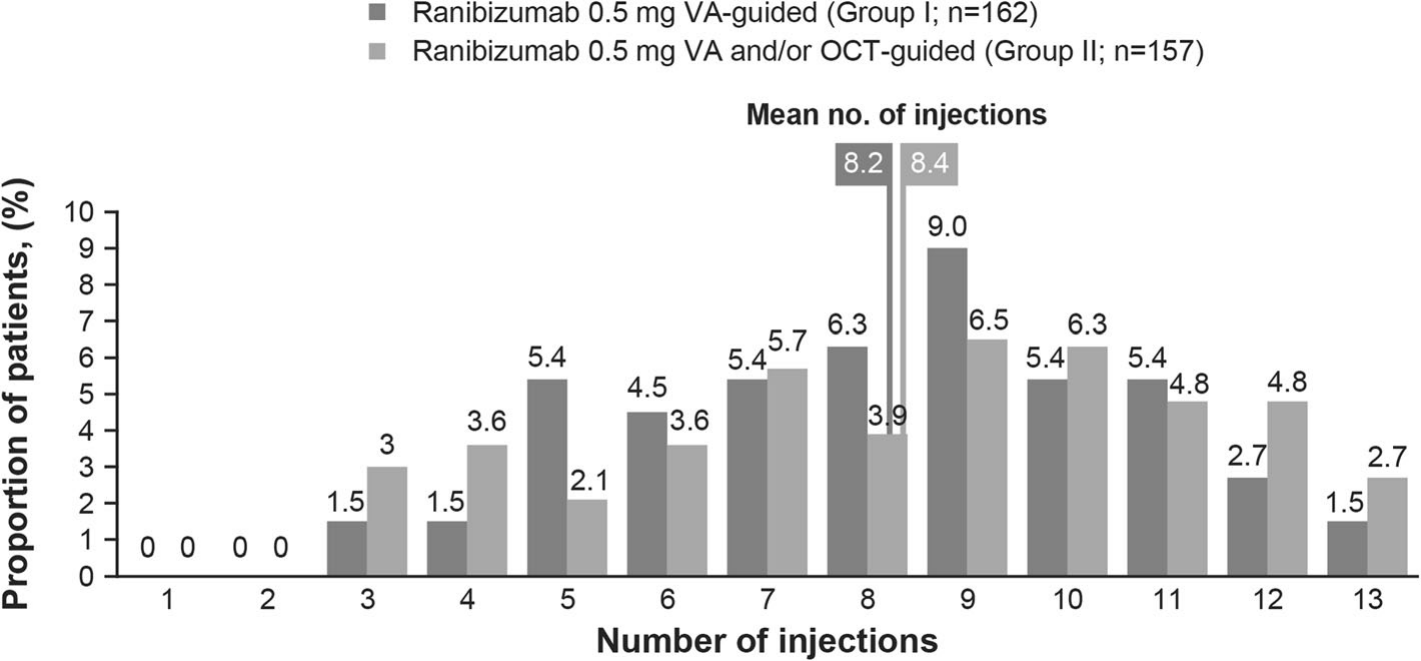
Treatment exposure. n, number of patients with observation period equal or longer than 12 months (Safety set); OCT, optical coherence tomography; VA, visual acuity

The mean (SD) number of injections was 8.2 (2.46) and 8.4 (2.87) in Groups I and II, respectively. The frequencies of ranibizumab injections administered over 12 months were generally comparable in both treatment groups (Fig. 5).

### Safety

Ocular serious AEs (SAEs) were reported in a small but comparable proportion of patients in both groups (Table 2). Non-ocular SAEs were reported in 8.1% of patients in Group I and in 12.8% of patients in Group II (Table 2). Seven patients died during the study; one patient in Group I (due to cardiopulmonary failure) and six patients in Group II (due to myocardial infarction, cardiovascular insufficiency, pneumonia, squamous cell carcinoma, congestive cardiac failure, and unknown causes [*n* = 1 each]). One death reported in Group II due to unknown causes was suspected to have a causal relationship with the study drug.

**Table 2:** Ocular and non-ocular serious AEs (safety set)

Preferred term, n (%)	Ranibizumab 0.5 mg VA only (Group I) *N* = 334	Ranibizumab 0.5 mg VA and/or OCT (Group II) *N* = 336
Ocular, Total	2 (0.6)	4 (1.2)
- VA reduced	1 (0.3)	1 (0.3)
- VA tests abnormal	1 (0.3)	0
- Choroidal hemorrhage	0	1 (0.3)
- Encephalitis post varicella	0	1 (0.3)
- Retinal artery embolism	0	1 (0.3)
Non-ocular, Total^a^	27 (8.1)	43 (12.8)
- Osteoarthritis	2 (0.6)	1 (0.3)
- Syncope	2 (0.6)	0
- Femoral neck fracture	1 (0.3)	4 (1.2)
- Lower respiratory tract infection	1 (0.3)	2 (0.6)
- Atrial fibrillation	0	3 (0.9)
- Hypertension	0	2 (0.6)
- Ovarian cyst	0	2 (0.6)
- Pneumonia	0	3 (0.9)

The safety set consisted of all patients who received at least one application of study drug and had at least one post-baseline safety assessment

MedDRA Version 18.1 was used for the reporting of AEs

A patient with multiple occurrences of an adverse event under one treatment was counted only once in the AEs category

Preferred terms are sorted by descending order of incidence in Group I column

*AE* adverse event, *MedDRA* Medical Dictionary for Regulatory Activities, *OCT* optical coherence tomography, *VA* visual acuity

^a^Events reported in ≥2 patients in any group. Treatment exposure: The mean (SD) number of injections for the overall population (safety set) was 7.3 (2.67) and 7.6 (2.98) in Groups I and II, respectively; the median number of injections was 7.0 in both groups

Overall, ocular and non-ocular AEs were reported in a comparable proportion of patients in both groups (Table 3). The ocular AE with the highest incidence in Group I was conjunctival hemorrhage (5.7%) and IOP increase in Group II (6.3%, Table 3). There were no cases of endophthalmitis.

**Table 3:** Ocular and non-ocular AEs^a^ (safety set)

Preferred term, n (%)	Ranibizumab 0.5 mg VA only (Group I) *N* = 334	Ranibizumab 0.5 mg VA and/or OCT (Group II) *N* = 336
Ocular, Total	105 (31.4)	106 (31.5)
- Conjunctival hemorrhage	19 (5.7)	10 (3.0)
- IOP increased	12 (3.6)	21 (6.3)
- Eye pain	10 (3.0)	9 (2.7)
- Dry eye	10 (3.0)	7 (2.1)
- VA reduced	7 (2.1)	1 (0.3)
- Vitreous floaters	6 (1.8)	8 (2.4)
Non-ocular, Total	156 (46.7)	143 (42.6)
- Nasopharyngitis	24 (7.2)	11 (3.3)
- Hypertension	18 (5.4)	16 (4.8)
- Influenza	15 (4.5)	15 (4.5)
- Cough	7 (2.1)	7 (2.1)
- Bronchitis	7 (2.1)	6 (1.8)
- Osteoarthritis	7 (2.1)	6 (1.8)
- Arthralgia	7 (2.1)	5 (1.5)
- Back pain	4 (1.2)	10 (3.0)
- Urinary tract infection	2 (0.6)	7 (2.1)

The safety set consisted of all patients who received at least one application of study drug and had at least one post-baseline safety assessment

MedDRA Version 18.1 was used for the reporting of AEs

A patient with multiple occurrences of an AE under one treatment was counted only once in the AEs category

Preferred terms are sorted by descending order of incidence in Group I column

*AE* adverse event, *IOP* intraocular pressure, *MedDRA* Medical Dictionary for Regulatory Activities, *OCT* optical coherence tomography, *VA* visual acuity

^a^Reported in ≥2% of patients in any group. Treatment exposure: The mean (SD) number of injections for the overall population (safety set) was 7.3 (2.67) and 7.6 (2.98) in Groups I and II, respectively; the median number of injections was 7.0 in both groups

Ocular and non-ocular AEs suspected to be related to the study drug and/or ocular injection were also reported in a comparable proportion of patients in both groups. Conjunctival hemorrhage in Group I and IOP in Group II were the most common ocular AEs (see, Additional file 6: Table S3). Ocular AEs leading to study drug discontinuation were reported by 2 patients, one in each group (Group 1: retinal detachment and VA reduced; Group II: vitreous hemorrhage). Nine patients discontinued from study drug due to non-ocular AEs, one from Group I (asthma) and eight from Group II (cardiac failure, presyncope, migraine, cholestasis, chronic myeloid leukemia, ischemic stroke, neutropenic sepsis, squamous cell carcinoma, and death [*n* = 1 each]).

## Discussion

The OCTAVE study demonstrates that a ranibizumab retreatment strategy based on VA alone (Group I) as well as VA and/or OCT (Group II) resulted in improved visual and anatomic outcomes. Numerically higher BCVA gains were observed in Group II suggesting that the use of VA and/or OCT may provide additional benefits over VA only-guided retreatment decisions, which is in line with clinical experience. Mirroring the BCVA findings, the mean decrease in CSFT at Month 12 was also numerically greater in Group II compared with Group I. Because the study was terminated early, the retreatment strategies could not be compared as planned. Nevertheless, the results are consistent with the current ranibizumab label [5], which recommends that retreatment decisions should be guided by disease activity assessment (both visual and anatomical parameters, including OCT). The suitability of an individualized treatment approach using both VA and OCT-guided retreatment criteria in nAMD patients was subsequently confirmed in a meta-analysis that showed sustained visual improvements over 2 years with this treatment regimen [16].

The ability of OCT to detect the earliest signs of disease activity with fluid re-accumulation in the macula, even before leakage detection on FA, was demonstrated in the extension study of the phase 1 and 2 studies with ranibizumab, in which OCT was used in conjunction with FA [8]. The PrONTO (Prospective Optical coherence tomography imaging of patients with Neovascular AMD Treated with intra-Ocular ranibizumab [Lucentis]) study, which was designed to evaluate an OCT-guided, variable ranibizumab-dosing regimen in patients with nAMD, demonstrated that a variable dosing regimen could result in visual and anatomic outcomes similar to the fixed-dosing phase 3 trials, with fewer injections, over a 2-year period [8, 10]. The study also demonstrated the usefulness of OCT to guide retreatment with ranibizumab. In clinical practice too, high levels of inter-observer agreement have been reported for the detection of macular fluid and other features of disease activity on OCT on a per patient basis [17], suggesting that OCT offers a more standardized and earlier detection of disease activity than VA alone in patients with nAMD. However, guidelines and evidence suggest that OCT and FA (which has been the reference standard) may both be needed for comprehensive diagnosis of active disease in patients with nAMD but OCT monitoring may suffice for follow-up and disease monitoring [13, 18].

The BCVA and CSFT improvements observed at Month 12 in OCTAVE were comparable to those reported in previous studies using VA and/or OCT-guided retreatment criteria with individualized or PRN ranibizumab [7, 19–21]. Other than a greater foveal thickness (mean CSFT > 500 μm vs 320 to 470 μm for other studies), patient baseline characteristics were comparable with these studies [7, 19–21]. This greater foveal thickness is explained by the prior definition for CSFT being measured from the Bruch’s membrane to the inner limiting membrane, whereas other studies may have measured from the retinal pigment epithelium (RPE) to the inner limiting membrane and thus exclude sub-RPE fluid from their CSFT. Treatment exposure and treatment patterns over time were similar with the two treatment regimens. The mean number of injections up to Month 12 in OCTAVE (8.2 in Group I and 8.4 in Group II) was similar to the mean number of injections reported in other controlled clinical studies using individualized or PRN treatment regimens for nAMD (6.5–8 injections) [19, 21], but was higher than those reported in real-world studies (4–6 injections) [22–26].

Specific qualitative morphologic parameters have an important role with respect to visual prognosis in patients with nAMD [27]. In OCTAVE, the presence of SRF at baseline had no effect on visual outcomes at 12 months; however, the gains were numerically higher in Group II. These findings are consistent with a subanalysis of the EXCITE trial that showed no effect of baseline SRF on later visual outcomes [11, 27]. A previous study reported better visual outcomes in eyes with subfoveal neovascular lesions than in those with extrafoveal CNV lesions [28]. Similarly, in our study, we observed a trend towards higher mean BCVA gains in patients without extrafoveal CNV lesions than those with extrafoveal CNV lesions. Anatomic outcomes at Month 12 were comparable between those with or without extrafoveal CNV lesions at baseline. The presence of cysts at baseline was associated with lower mean BCVA gains from baseline at Month 12 in both groups (exploratory *p* < 0.05 in both groups). Lower mean VA gain in nAMD patients with cysts at baseline has also been reported in a previous study [29]. There was a slight indication that patients with baseline cysts achieved higher BCVA gains in Group II, as compared to Group I, while the gains for patients without baseline cysts were similar. Out of patients with cysts, those with cysts larger than 400 μm had the highest BCVA gain and CSFT reduction at Month 12 compared with those with small or medium cysts.

Overall, the safety observations were in line with the well-established safety profile of ranibizumab. There were no cases of endophthalmitis and no new safety findings in the study. Although a higher number of deaths were reported in Group II, all except one were considered by investigators to be unrelated to ranibizumab treatment.

The primary limitation of the study was the premature termination of the study, which limited the available pool of data to assess the primary objectives of the study. Efficacy results were presented for a selected subset, i.e. the patients who completed 12 months. Subset selection can be associated with a selection bias, However, in OCTAVE, for 94% of the patients the time in the study was determined by an administrative event, the early study termination. The selection bias was therefore expected to be minimal and was not evident from the demographic and baseline characteristics of the entire study population versus the subset of patients who completed 12 months. Additional limitations are the evaluating and/or treating investigator not being masked to retreatment criteria, and the availability of OCT results in Group I which might have influenced at least some retreatment decisions. This could have led to smaller differences between the two groups. Further, the decision to retreat with ranibizumab was solely at the investigator’s discretion and not based on defined parameters, which, although reflective of real-world clinical practice, could have resulted in inconsistencies across investigative sites.

## Conclusions

Ranibizumab treatment resulted in visual and anatomic gains at 12 months for both retreatment strategies, with a trend for more favorable outcomes in the group combining objective morphological OCT criteria with VA data to guide re-treatment decisions. There were no new safety signals in OCTAVE. The results were in agreement with the well-established safety profile of ranibizumab.

## Supplementary information

**Additional file 1: Table S1.** List of Independent Ethics Committees (IECs) or Institutional Review Boards (IRBs).

**Additional file 2.** Exclusion criteria.

**Additional file 3: Figure S1.** BCVA and CSFT outcomes at Month 12 stratified by presence of extrafoveal CNV lesions at baseline (FAS).

**Additional File 4: Figure S2.** BCVA and CSFT outcomes at Month 12 stratified by (A) presence of SRF, (B) presence of cysts and (C) thickness of cysts at baseline, for patients with month 12 BCVA data (FAS).

**Additional file 5: Table S2.** Linear regression model for change in BCVA from baseline to Month 12 (FAS).

**Additional file 6: Table S3.** Ocular and non-ocular AEs suspected to be related to study drug and/or injection procedure (safety set).

## Data Availability

Most data generated or analyzed during this study are included in this article [and its supplementary information files]. Additional datasets used and/or analyzed during the current study are available on reasonable request.

## Abbreviations

AEs: Adverse events
Anti-VEGF: Anti-vascular endothelial growth factor
BCVA: Best-corrected visual acuity
CNV: Choroidal neovascularization
CSFT: Central subfield thickness.
ETDRS: Early Treatment Diabetic Retinopathy Study
FA: Fluorescein angiography
IRF: Intraretinal fluid
IOP: Intraocular pressure
nAMD: Neovascular age-related macular degeneration
OCT: Optical coherence tomography
OCTAVE: OCT And Vision Evaluation study
PRN: Pro re nata
RPE: Retinal pigment epithelium
SD: Standard deviation
SRF: Subretinal fluid
VA: Visual acuity
VRC: Vienna Reading Center
