# Supplementary material for: Functional versus functional and anatomical criteria-guided ranibizumab treatment in patients with neovascular age-related macular degeneration – results from the randomized, phase IIIb OCTAVE study
Source: BMC Ophthalmol. 2020 Jan 9;20:18. doi: 10.1186/s12886-019-1251-6 (PMC6953154; doi:10.1186/s12886-019-1251-6)
Supplement: Supplementary file 1 — Additional file 1: Table S1. List of Independent Ethics Committees (IECs) or Institutional Review Boards (IRBs). [file 12886_2019_1251_MOESM1_ESM.docx]

**Additional file 1 Table S1**. **List of Independent Ethics Committees (IECs) or Institutional Review Boards (IRBs)**

| **Center No.** | **Ethics Committee or Institutional Review Board** | **Department / Organization** | **Address Country** |
| --- | --- | --- | --- |
| 0001 | Stamboulian | Comité de Etica en Investigacion Clinica | CABA Buenos |
|  |  | (CEIC) | Aires C1117ABK |
|  |  |  | Argentina |
| 0002 | Stamboulian | Comité de Etica en Investigacion Clinica | CABA Buenos |
|  |  | (CEIC) | Aires C1117ABK |
|  |  |  | Argentina |
| 0015 | Ethikkommission Medizinische |  | Vienna 1090 |
|  | Universität Wien |  | Austria |
| 0035 | Capital Health District Authority |  | Halifax Nova |
|  | Ethic committee |  | Scotia B3H 1V7 |
|  |  |  | Canada |
| 0037 | IRB Services |  | Aurora Ontario |
|  |  |  | L4G 0A5 |
|  |  |  | Canada |
| 0038 | Western University Health |  | London Ontario |
|  | Science Research Ethics Board |  | N6G 1G9 |
|  |  |  | Canada |
| 0041 | Ottawa Health Science Network |  | Ottawa Ontario |
|  | Research Ethics Board |  | K1Y 4E9 |
|  |  |  | Canada |
| 0042 | The University of British |  | Vancouver British |
|  | Columbia Office of Research |  | Columbia V5Z 1L8 |
|  | Services, Clinical Research |  | Canada |
|  | Ethics Board |  |  |
| 0044 | IRB Services |  | Aurora Ontario |
|  |  |  | L4G 0A5 |
|  |  |  | Canada |
| 0055 | CREIMED |  | Medellin Antioquia |
|  |  |  | Colombia |
| 0058 | Comité de ética en Investigación |  | Bogotá |
|  | de la Fundación oftalmologica |  | Cundinamarca |
|  | Nacional |  | Colombia |
| 0070 | MEK Fakultní nemocnice Hradec |  | Hradec Králové |

500 05

| **Center No.** | **Ethics Committee or Institutional Review Board** | **Department / Organization** | **Address Country** |
| --- | --- | --- | --- |
|  | Králové |  | Czech Republic |
| 0071 | Etická komise Fakultní |  | Praha 10 100 34 |
|  | nemocnice Královské Vinohrady |  | Czech Republic |
| 0072 | Etická komise Fakultní |  | Brno 625 00 |
|  | nemocnice Brno |  | Czech Republik |
| 0073 | Etická komise FN a LF UP |  | Olomouc 775 20 |
|  | Olomouc |  | Czech Republic |
| 0080 | HUS eettiset toimikunnat | Operatiivinen eettinen toimikunta | Helsinki 00029 |
|  |  |  | HUS |
|  |  |  | Finland |
| 0081 | HUS eettiset toimikunnat | Operatiivinen eettinen toimikunta | Helsinki 00029 |
|  |  |  | HUS |
|  |  |  | Finland |
| 0090 | CPP Ile de France III | Hôpital Tarnier Cochin | Paris 75006 |
|  |  | 89 rue d'Assas | France |
| 0100 | Ethikkommission der | Medizinische Fakültät der Westfälischen | Muenster 48147 |
|  | Ärztekammer Westfalen-Lippe | Wilhelms Universität Muenster | Germany |
| 0101 | Landesärztekammer Hessen | Ethikkommission | Frankfurt am Main |
|  |  |  | 60488 |
|  |  |  | Germany |
| 0102 | Sächsische Landesärztekammer | Ethik-Kommission | Dresden 01099 |
|  |  |  | Germany |
| 0103 | Ethikkommission der | Medizinische Fakültät der Westfälischen | Muenster 48147 |
|  | Ärztekammer Westfalen-Lippe | Wilhelms Universität Muenster | Germany |
| 0104 | Ethikkommission an der | Klinikum der Universität Regensburg | Regensburg |
|  | Universität Regensburg |  | 93053 |
|  |  |  | Germany |
| 0105 | Albert-Ludwig-Universität | Ethikkommission | Freiburg 79106 |
|  | Freiburg |  | Germany |
| 0106 | Rheinische Friedrich-Wilhelms- | Medizinische Fakultät Bonn / | Bonn 53105 |
|  | Universität Ethik-Kommission | Biomedizinisches Zentrum | Germany |
| 0107 | Geschäftsstelle Ethikkommission | Universität zu Köln | Köln 50937 |
|  |  |  | Germany |
| 0111 | Ludwig-Maximilian-Universität | Ethik-Kommission | München 80336 |
|  | München / Klinikum der |  | Germany |
|  | Universität |  |  |
| 0125 | Scientific Council of General | N/A | Athens N/A 11526 |
|  | Hospital of Athens "Korgialenio- |  | Greece |
|  | Benakio" |  |  |
| 0126 | Scientific Council of University | N/A | Ioannina N/A |
|  | General Hospital of Ioannina |  | 45500 |
|  |  |  | Greece |
| 0127 | Scientific Council of General | N/A | Athens N/A 11527 |
|  | Hospital of Athens, "G. |  | Greece |
|  | Gennimatas" |  |  |
| 0129 | Scientific Council of Athens Eye | N/A | Glyfada N/A 16675 |
|  | Hospital |  | Greece |

| **Center No.** | **Ethics Committee or Institutional Review Board** | **Department / Organization** | **Address Country** |
| --- | --- | --- | --- |
| 0141 | Comité de Etica Independiente | Independent | Guatemala City |
|  | ZUGUEME |  | Guatemala |
|  |  |  | Guatemala |
| 0142 | Comité de Etica Independiente | Independent | Guatemala City |
|  | ZUGUEME |  | Guatemala |
|  |  |  | Guatemala |
| 0155 | Medical Research Council |  | Budapest, Arany |
|  | /Ethics Committee for Clinical |  | János utca 6-8, |
|  | Pharmacology |  | 1051 |
|  |  |  | Hungary |
| 0156 | Medical Research Council |  | Budapest, Arany |
|  | /Ethics Committee for Clinical |  | János utca 6-8, |
|  | Pharmacology |  | 1051 |
|  |  |  | Hungary |
| 0157 | Medical Research Council |  | Budapest, Arany |
|  | /Ethics Committee for Clinical |  | János utca 6-8, |
|  | Pharmacology |  | 1051 |
|  |  |  | Hungary |
| 0158 | Medical Research Council |  | Budapest, Arany |
|  | /Ethics Committee for Clinical |  | János utca 6-8, |
|  | Pharmacology |  | 1051 |
|  |  |  | Hungary |
| 0159 | Medical Research Council |  | Budapest, Arany |
|  | /Ethics Committee for Clinical |  | János utca 6-8, |
|  | Pharmacology |  | 1051 |
|  |  |  | Hungary |
| 0160 | Medical Research Council |  | Budapest, Arany |
|  | /Ethics Committee for Clinical |  | János utca 6-8, |
|  | Pharmacology |  | 1051 |
|  |  |  | Hungary |
| 0161 | Medical Research Council |  | Budapest, Arany |
|  | /Ethics Committee for Clinical |  | János utca 6-8, |
|  | Pharmacology |  | 1051 |
|  |  |  | Hungary |
| 0162 | Medical Research Council |  | Budapest, Arany |
|  | /Ethics Committee for Clinical |  | János utca 6-8, |
|  | Pharmacology |  | 1051 |
|  |  |  | Hungary |
| 0163 | Medical Research Council |  | Budapest, Arany |
|  | /Ethics Committee for Clinical |  | János utca 6-8, |
|  | Pharmacology |  | 1051 |
|  |  |  | Hungary |
| 0175 | St Vincent's Healthcare Group | Elm Park | Dublin Dublin 4 |
|  | Ltd |  | ROI |
| 0176 | St Vincent's Healthcare Group | Elm Park | Dublin Dublin 4 |
|  | Ltd |  | ROI |
| 0190 | COMITATO ETICO DELL'IRCCS |  | MILANO MILANO |
|  | FONDAZIONE SAN RAFFAELE |  | 20132 |
|  | DEL MONTE TABOR |  | ITALY |

| **Center No.** | **Ethics Committee or Institutional Review Board** | **Department / Organization** | **Address Country** |
| --- | --- | --- | --- |
| 0191 | COMITATO ETICO LOCALE | AZIENDA OSPEDALIERA OSPEDALE | MILANO MILANO |
|  | PER LA SPERIMENTAZIONE | LUIGI SACCO | 20157 |
|  | CLINICA |  | ITALY |
| 0192 | COMITATO ETICO AZIENDALE | AZIENDA OSPEDALIERO- | UDINE UDINE |
|  |  | UNIVERSITARIA S. MARIA DELLA | 33100 |
|  |  | MISERICORDIA | ITALY |
| 0194 | COMITATO ETICO AREA | AZIENDA OSPEDALIERO- | FIRENZE |
|  | VASTA CENTRO | UNIVERSITARIA CAREGGI | FIRENZE 50134 |
|  |  |  | ITALY |
| 0205 | Lithuanian Bioethics Committee, | NA | Vilnius NA 01402 |
|  | Vilnius str.16 |  | Lithuania |
| 0206 | Lithuanian Bioethics Committee, | NA | Vilnius NA 01402 |
|  | Vilnius str.16 |  | Lithuania |
| 0216 | COMITÉ DE ÉTICA EN |  | Mexico Mexico |
|  | INVESTIGACIÓN DEL |  | 06800 |
|  | INSTITUTO DE |  | Mexico |
|  | OFTALMOLOGIA “FUNDACIÓN |  |  |
|  | DE ASISTENCIA PRIVADA |  |  |
|  | CONDE DE VALENCIANA IAP” |  |  |
| 0225 | cEC Radboud Hosptital/ CCMO | CMO Arnhem Nijmegen | Nijmegen |
|  |  |  | Nijmegen 6500 HB |
|  |  |  | Netherlands |
| 0226 | cEC Radboud Hosptital/ CCMO | CMO Arnhem Nijmegen | Nijmegen |
|  |  |  | Nijmegen 6500 HB |
|  |  |  | Netherlands |
| 0228 | cEC Radboud Hosptital/ CCMO | CMO Arnhem Nijmegen | Nijmegen |
|  |  |  | Nijmegen 6500 HB |
|  |  |  | Netherlands |
| 0240 | Comité de Bioética de la | Independent | Panama City |
|  | Investigación - Instituto |  | Panama NA |
|  | Conmemorativo Gorgas de |  | Panama |
|  | Estudios de la Salud (CIB- |  |  |
|  | ICGGES) |  |  |
| 0300 | CEIC |  | Lisboa Lisboa |
|  |  | CEIC - Parque da Saúde de Lisboa | 1749-004 |
|  |  | Av. do Brasil, 53 - Pav. 17-A | Portugal |
| 0301 | CEIC |  | Lisboa Lisboa |
|  |  | CEIC - Parque da Saúde de Lisboa | 1749-004 |
|  |  | Av. do Brasil, 53 - Pav. 17-A | Portugal |
| 0302 | CEIC |  | Lisboa Lisboa |
|  |  | CEIC - Parque da Saúde de Lisboa | 1749-004 |
|  |  | Av. do Brasil, 53 - Pav. 17-A | Portugal |
| 0400 | Eticka komisia, | OFTAL s.r.o. | Zvolen Slovakia |
|  |  |  | 96001 |
|  |  |  | Slovakia |
| 0401 | Eticka komisia, | Fakultna nemocnica Trencin | Trencin Slovakia |
|  |  |  | 91171 |
|  |  |  | Slovakia |

### Center Ethics Committee or Department / Organization Address

**No.**

**Institutional Review Board**

**Country**

0402 Eticka komisia, FNsP Zilina Zilina Slovakia 01207

Slovakia

0403 Eticka komisia, Univerzitna nemocnica sv. Cyrila a Metoda Bratislava Slovakia

85107

Slovakia

0404 Eticka komisia, Fakultna nemocnica Nitra Nitra Slovakia

95001

Slovakia

0405 Eticka komisia, Univerzitná nemocnica Bratislava Bratislava Slovakia

82606

Slovakia

0500 Comité Etico de Investigación Clínica de Asturias

0501 Comité Ético de Investigación Clínica de Galicia. Consellería de Sanidade

0503 Secretaría del Comite Etico de Investigación Clinica. Dirección de Farmacia. Dep Sanidad Govierno Vasco

0505 CEIC Hospital de L`Hospitalet.

Consorci Sanitari Integral

C/Celestino Villamil s/n planta 5 Oviedo Asturias

33006

Spain

C/San Lazaro s/n Santiago de Compostela A Coruña 15703 Spain

C/Donostia San Sebastian n. 1 Vitoria-Gasteiz Araba 01010 Spain

C/Joseph Molins 29-41 L´Hospitalet de Llobregat Barcelona 08906 Spain

0600 Regionala etikprövningsnämnden Linköping 58183 Sweden

0701 Kantonale Ethikkommission Zürich

0702 Kantonale Ethikkommission Bern (KEK)

0703 Commission cantonale d'éthique de la recherche sur l'être humain

0704 Commission cantonale d'éthique de la recherche CCER

0800 Hacettepe University Clinical Trials Ethics Committee

0801 Hacettepe University Clinical Trials Ethics Committee

0802 Hacettepe University Clinical Trials Ethics Committee

0820 NRES Committee London - South East

0822 NRES Committee London - South East

Zuerich 8090 Switzerland

Bern 3010 Switzerland

Lausanne 1012 Switzerland

Genève 1207 Switzerland

Ankara 06100 Turkey

Ankara 06100 Turkey

Ankara 06100 Turkey

Bristol Research Ethics Committee Centre Bristol BS1 2NT

UK

Bristol Research Ethics Committee Centre Bristol BS1 2NT

UK

| **Center No.** | **Ethics Committee or Institutional Review Board** | **Department / Organization** | **Address Country** |
| --- | --- | --- | --- |
| 0823 | NRES Committee London - South East | Bristol Research Ethics Committee Centre | Bristol BS1 2NT UK |
| 0824 | NRES Committee London - South East | Bristol Research Ethics Committee Centre | Bristol BS1 2NT UK |
| 0825 | NRES Committee London - South East | Bristol Research Ethics Committee Centre | Bristol BS1 2NT UK |
| 0826 | NRES Committee London - South East | Bristol Research Ethics Committee Centre | Bristol BS1 2NT UK |
| 0827 | NRES Committee London - South East | Bristol Research Ethics Committee Centre | Bristol BS1 2NT UK |
| 0828 | NRES Committee London - South East | Bristol Research Ethics Committee Centre | Bristol BS1 2NT UK |
| 0829 | NRES Committee London - South East | Bristol Research Ethics Committee Centre | Bristol BS1 2NT UK |
| 0830 | NRES Committee London - South East | Bristol Research Ethics Committee Centre | Bristol BS1 2NT UK |
| 0831 | NRES Committee London - South East | Bristol Research Ethics Committee Centre | Bristol BS1 2NT UK |
| 0832 | NRES Committee London - South East | Bristol Research Ethics Committee Centre | Bristol BS1 2NT UK |
| 0833 | NRES Committee London - South East | Bristol Research Ethics Committee Centre | Bristol BS1 2NT UK |
| 0834 | NRES Committee London - South East | Bristol Research Ethics Committee Centre | Bristol BS1 2NT UK |
| 0835 | NRES Committee London - South East | Bristol Research Ethics Committee Centre | Bristol BS1 2NT UK |
| 0836 | NRES Committee London - South East | Bristol Research Ethics Committee Centre | Bristol BS1 2NT UK |
