# Supplementary material for: Functional versus functional and anatomical criteria-guided ranibizumab treatment in patients with neovascular age-related macular degeneration – results from the randomized, phase IIIb OCTAVE study
Source: BMC Ophthalmol. 2020 Jan 9;20:18. doi: 10.1186/s12886-019-1251-6 (PMC6953154; doi:10.1186/s12886-019-1251-6)
Supplement: Supplementary file 2 — Additional file 2. Exclusion criteria. [file 12886_2019_1251_MOESM2_ESM.docx]

**Additional File 2**

Exclusion criteria

Patients fulfilling any of the following criteria were not eligible for inclusion in the study. No additional exclusions could be applied by the investigator in order to ensure that the study population was representative of all eligible patients.

*Exclusion criteria for patients*

1. Inability to comply with study or follow-up procedures

2. Pregnant or nursing (lactating) women, where pregnancy was defined as the state of a female after conception and until the termination of gestation, confirmed by a positive h-chorionic gonadotropin laboratory test

3. Women of child-bearing potential, defined as all women physiologically capable of becoming pregnant, unless they were using effective methods of contraception during dosing of study drug. Effective contraception methods included: Total abstinence (when this was in line with the preferred and usual lifestyle of the patient). Periodic abstinence (e.g., calendar, ovulation, symptothermal,
post-ovulation methods) and withdrawal were not acceptable methods of contraception

- Female sterilization (had surgical bilateral oophorectomy with or without hysterectomy) or tubal ligation at least 6 weeks before taking study drug. In case of oophorectomy alone, only when the reproductive status of the woman had been confirmed by follow-up hormone level assessment
- Male sterilization (at least 6 months prior to screening). For female patients on the study, the vasectomized male partner was to be her sole partner
- Barrier methods of contraception: condom or occlusive cap (diaphragm or cervical/vault caps) with spermicidal foam/gel/film/cream/vaginal suppository
- Use of oral, injected, or implanted hormonal methods of contraception or other forms of hormonal contraception that had comparable efficacy (failure rate <1%), for example hormone vaginal ring or transdermal hormone contraception
- Placement of an intrauterine device or intrauterine system

In the case of oral contraception use, women were to be stable on the same contraceptive pill for a minimum of 3 months before taking study drug. Women were considered post-menopausal and not of childbearing potential if they had 12 months of natural (spontaneous) amenorrhea with an appropriate clinical profile (e.g., age appropriate, history of vasomotor symptoms) or had surgical bilateral oophorectomy (with or without hysterectomy) or tubal ligation at least 6 weeks previously. In the case of oophorectomy alone, only when the reproductive status of the woman had been confirmed by follow-up hormone level assessment was she considered not of childbearing potential

*Exclusion criteria for systemic medical history and conditions*

4. Any type of systemic disease or its treatment, including any medical condition (controlled or uncontrolled) that could be expected to progress, recur, or change to such an extent that it could bias the assessment of the clinical status of the patient to a significant degree or put the patient at special risk

5. Stroke or myocardial infarction less than 3 months prior to screening

6. Uncontrolled blood pressure defined as a systolic value of >160 mm Hg or diastolic value of >100 mm Hg at screening or baseline

7. Known hypersensitivity to ranibizumab or any component of the ranibizumab formulation, or fluorescein

*Exclusion criteria for ocular medical history and conditions*

For either eye:

8. Any active periocular or ocular infection or inflammation (e.g., blepharitis, conjunctivitis, keratitis, scleritis, uveitis, endophthalmitis) at the time of screening or baseline Uncontrolled glaucoma (IOP ≥30 mm Hg on medication or according to investigator’s judgment) at the time of screening or baseline

10. Neovascularization of the iris or neovascular glaucoma at the time of screening or baseline

11. Inability to obtain OCT images of sufficient quality to be analyzed

For the study eye:

12. Cataract (if causing significant visual impairment), aphakia, severe vitreous hemorrhage, rhegmatogenous retinal detachment, proliferative retinopathy or choroidal neovascularization of any other cause than nAMD (e.g., ocular histoplasmosis, pathologic myopia) at the time of screening or baseline

13. Irreversible structural damage within 0.5 disc diameter of the center of the macula
(e.g., vitreomacular traction, epiretinal membrane, scar, laser burn, macular hole) at the time of screening or baseline that in the investigator’s opinion could substantially impact visual function improvement with treatment

14. Atrophy or fibrosis involving the center of the fovea

15. Total area of fibrosis comprising more than 50% of the lesion area

*Exclusion criteria for prior or current systemic medication*

16. Use of other investigational drugs within 30 days or 5 half-lives from baseline, whichever was longer

17. Use of any systemic anti-VEGF drugs within 3 months prior to baseline (e.g.,bevacizumab [Avastin^®^])

18. Use of systemic corticosteroids for at least 30 consecutive days within 3 months prior to

screening

19. Current or planned use of systemic medications known to be toxic to the lens, retina, or optic nerve, including deferoxamine, chloroquine/hydroxychloroquine (Plaquenil^®^), tamoxifen, phenothiazines, and ethambutol

*Exclusion criteria for prior or current ocular treatment*

For the fellow eye:

20. Treatment with any anti-angiogenic drugs (including any anti-VEGF agents) within 3 months prior to baseline in either eye (e.g., bevacizumab [Avastin^®^])

For the study eye:

21. Any intraocular procedure (including Yttrium-Aluminum-Garnet capsulotomy) within 2 months prior to baseline or anticipated within the next 6 months following baseline

22. Topical ocular corticosteroids administered for at least 30 consecutive days within 3 months prior to screening
