# Supplementary figures and images for: Functional versus functional and anatomical criteria-guided ranibizumab treatment in patients with neovascular age-related macular degeneration – results from the randomized, phase IIIb OCTAVE study
Source: BMC Ophthalmol. 2020 Jan 9;20:18. doi: 10.1186/s12886-019-1251-6 (PMC6953154; doi:10.1186/s12886-019-1251-6)

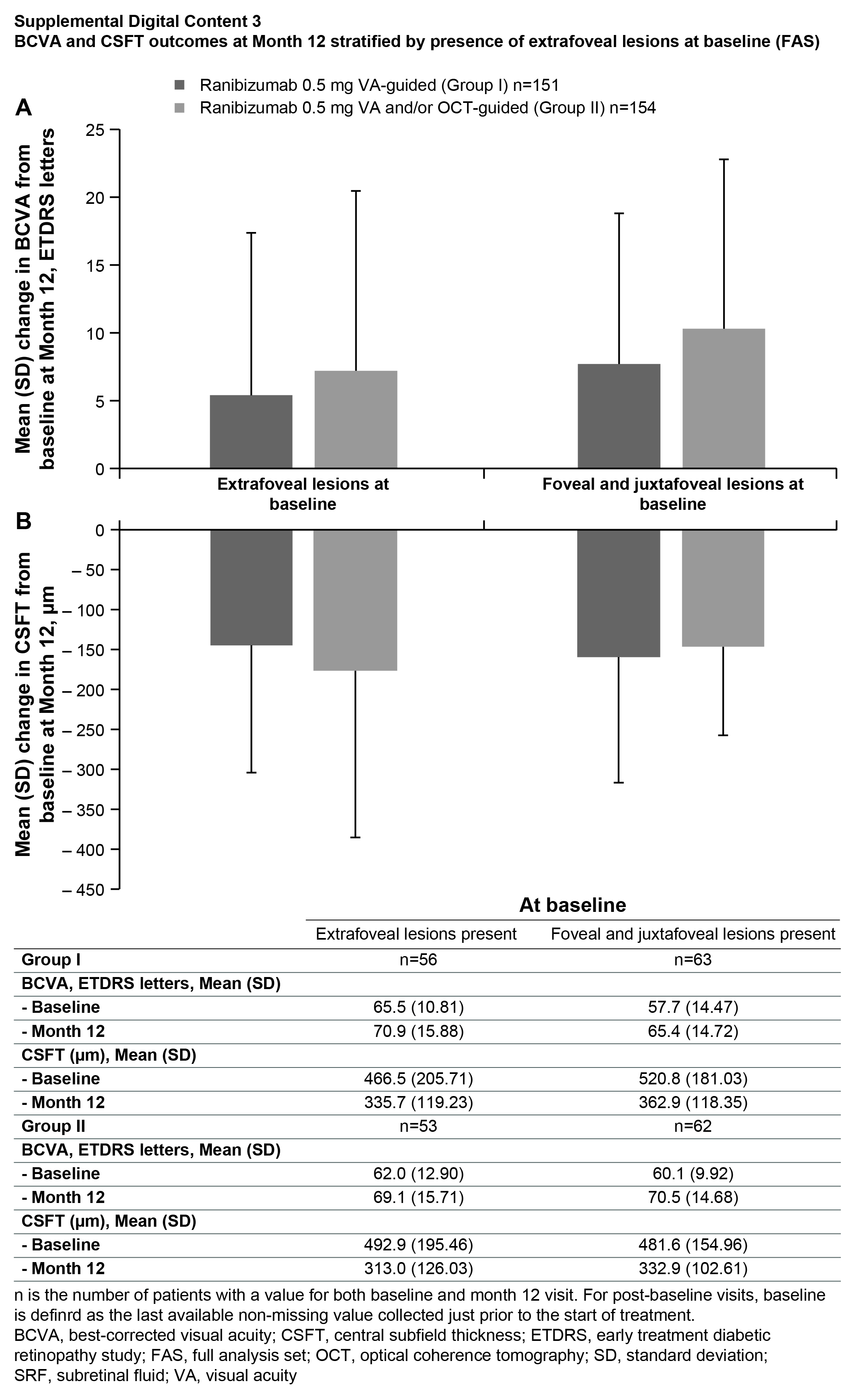

Supplement: Supplementary file 3 — Additional file 3: Figure S1. BCVA and CSFT outcomes at Month 12 stratified by presence of extrafoveal CNV lesions at baseline (FAS). [file 12886_2019_1251_MOESM3_ESM.tif]

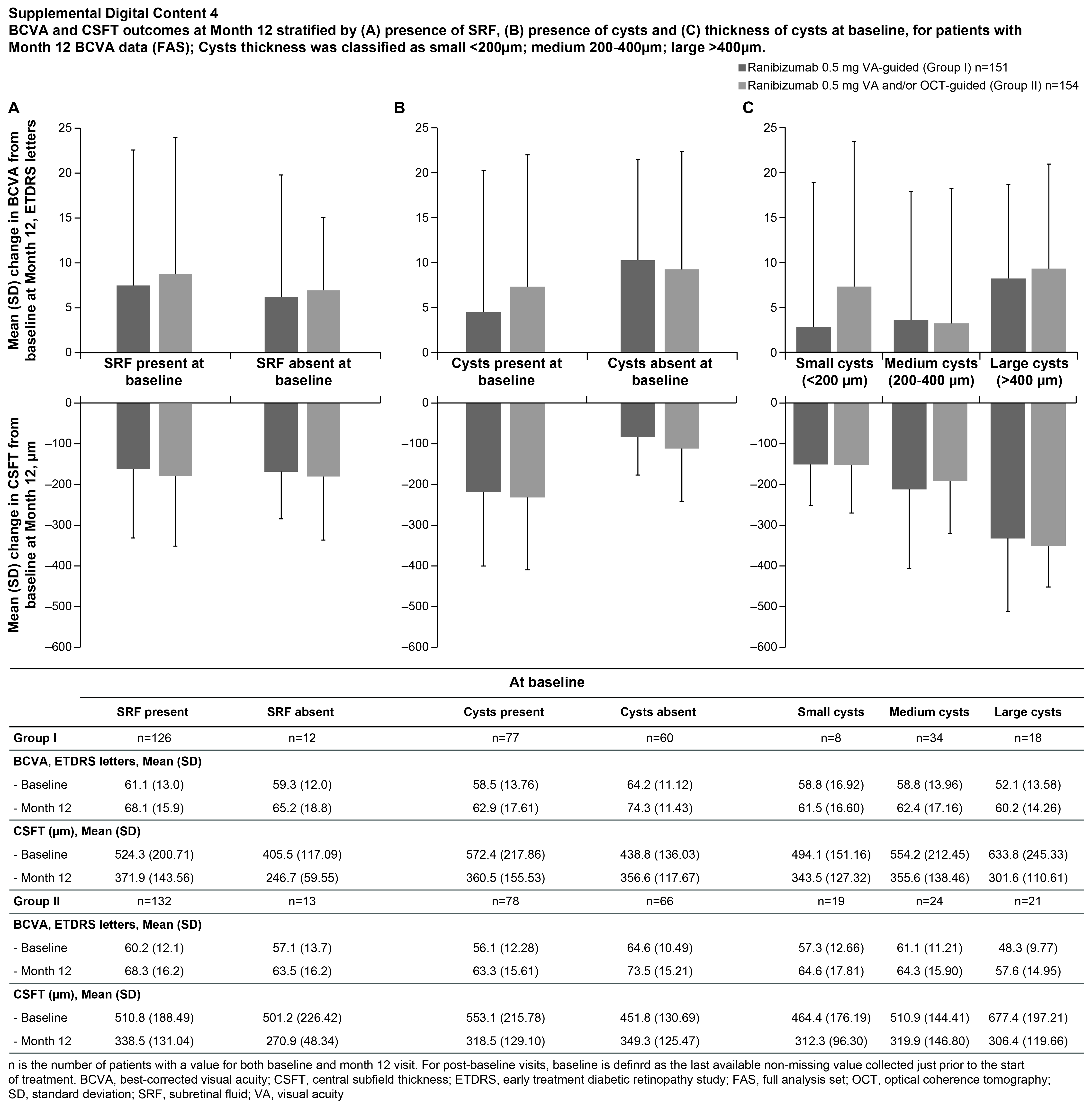

Supplement: Supplementary file 4 — Additional File 4: Figure S2. BCVA and CSFT outcomes at Month 12 stratified by (A) presence of SRF, (B) presence of cysts and (C) thickness of cysts at baseline, for patients with month 12 BCVA data (FAS). [file 12886_2019_1251_MOESM4_ESM.tif]
