# Supplementary material for: Functional versus functional and anatomical criteria-guided ranibizumab treatment in patients with neovascular age-related macular degeneration – results from the randomized, phase IIIb OCTAVE study
Source: BMC Ophthalmol. 2020 Jan 9;20:18. doi: 10.1186/s12886-019-1251-6 (PMC6953154; doi:10.1186/s12886-019-1251-6)
Supplement: Supplementary file 5 — Additional file 5: Table S2. Linear regression model for change in BCVA from baseline to Month 12 (FAS). [file 12886_2019_1251_MOESM5_ESM.docx]

**Additional file 5 Table S2**Linear regression model for change in BCVA from baseline to Month 12 (FAS)

| **Treatment** | **Baseline cysts** | **Mean change in BCVA (95% CI)** | **Difference  (95% CI)** | **p-value** |
| --- | --- | --- | --- | --- |
| Ranibizumab 0.5mg: VA only (Group I) | No | 11.37 (8.00, 14.75) | 7.64 (3.12, 12.16) | 0.0010 |
|  | Yes | 3.73 (0.77, 6.70) |  |  |
| Ranibizumab 0.5mg: VA and/or OCT (Group II) | No | 10.49 (7.26, 13.72) | 4.80 (0.34, 9.26) | 0.0350 |
|  | Yes | 5.69 (2.71, 8.67) |  |  |

Number of patients with non-missing covariate and Month 12 BCVA data. Difference of the mean change in BCVA is No Presence of cysts – Presence of cysts

Linear model of change in BCVA from baseline at Month 12 adjusting for treatment, baseline cysts, baseline BCVA and baseline cysts/treatment interaction

The FAS comprised all patients to whom a treatment regimen had been assigned

BCVA, best-corrected visual acuity; FAS, full analysis set; CI, confidence interval; OCT, optical coherence tomography; VA, visual acuity
