# Supplementary material for: Functional versus functional and anatomical criteria-guided ranibizumab treatment in patients with neovascular age-related macular degeneration – results from the randomized, phase IIIb OCTAVE study
Source: BMC Ophthalmol. 2020 Jan 9;20:18. doi: 10.1186/s12886-019-1251-6 (PMC6953154; doi:10.1186/s12886-019-1251-6)
Supplement: Supplementary file 6 — Additional file 6: Table S3. Ocular and non-ocular AEs suspected to be related to study drug and/or injection procedure (safety set). [file 12886_2019_1251_MOESM6_ESM.docx]

**Additional file 6 Table S3**

Ocular and non-ocular AEs suspected to be related to study drug and/or injection procedure (safety set)

| Preferred term, n (%) | Ranibizumab 0.5 mg VA only  (Group I) N=334 | Ranibizumab 0.5 mg VA and/or OCT  (Group II) N=336 |
| --- | --- | --- |
| **Ocular, Total** | 52 (15.6) | 53 (15.8) |
| Conjunctival hemorrhage | 19 (5.7) | 9 (2.7) |
| IOP increased | 7 (2.1) | 21 (6.3) |
| Eye pain | 7 (2.1) | 5 (1.5) |
| Vitreous floaters | 6 (1.8) | 5 (1.5) |
| Conjunctival hyperemia | 1 (0.3) | 3 (0.9) |
| Punctate keratitis | 1 (0.3) | 3 (0.9) |
| Retinal pigment epithelial tear | 2 (0.6) | 2 (0.6) |
| Corneal erosion | 2 (0.6) | 1 (0.3) |
| Injection site pain | 3 (0.9) | 0 |
| Ocular hypertension | 2 (0.6) | 1 (0.3) |
| SRF | 1 (0.3) | 2 (0.6) |
| Injection site erythema | 2 (0.6) | 0 |
| Injection site hemorrhage | 1 (0.3) | 1 (0.3) |
| nAMD | 1 (0.3) | 1 (0.3) |
| Ocular hyperemia | 1 (0.3) | 1 (0.3) |
| Vision blurred | 0 | 2 (0.6) |
| Blepharospasm | 0 | 1 (0.3) |
| Choroidal dystrophy | 1 (0.3) | 0 |
| Corneal epithelium defect | 0 | 1 (0.3) |
| Dry eye | 1 (0.3) | 0 |
| Eye discharge | 0 | 1 (0.3) |
| Eye hemorrhage | 0 | 1 (0.3) |
| Eye irritation | 0 | 1 (0.3) |
| Eye swelling | 0 | 1 (0.3) |
| Eyelid edema | 1 (0.3) | 0 |
| Macular hole | 0 | 1 (0.3) |
| Metamorphopsia | 0 | 1 (0.3) |
| Retinal depigmentation | 0 | 1 (0.3) |
| Retinal hemorrhage | 1 (0.3) | 0 |
| **Non-ocular, Total** | 10 (3.0) | 7 (2.1) |
| Hypertension | 4 (1.2) | 0 |
| Alopecia | 1 (0.3) | 0 |
| Angina pectoris | 1 (0.3) | 0 |
| Arthralgia | 0 | 1 (0.3) |
| Cardiovascular disorder | 1 (0.3) | 0 |
| Colitis ischemic | 1 (0.3) | 0 |
| Death | 0 | 1 (0.3) |
| Disorientation | 1 (0.3) | 0 |
| Epistaxis | 0 | 1 (0.3) |
| Headache | 0 | 1 (0.3) |
| Memory impairment | 1 (0.3) | 0 |
| Sleep disorder | 0 | 1 (0.3) |
| Syncope | 1 (0.3) | 0 |
| Thrombophlebitis | 0 | 1 (0.3) |
| Transient ischemic attack | 0 | 1 (0.3) |

The safety set consisted of all patients who received at least one application of study drug and had at least one
post-baseline safety assessment

MedDRA Version 18.1 was used for the reporting of AEs

A patient with multiple occurrences of an adverse event under one treatment was counted only once in the AEs category

AEs with start date on or after the date of first administration of study drug were counted

Preferred terms are sorted by descending order of incidence by total column
AE, adverse event; IOP, intraocular pressure; MedDRA, Medical Dictionary for Regulatory Activities; nAMD, neovascular age-related macular degeneration; OCT, optical coherence tomography; RBZ, ranibizumab; VA, visual acuity
